# The impact of insufficient resources on the quality-of-service delivery at a primary healthcare clinic in Limpopo

**DOI:** 10.4102/curationis.v48i1.2696

**Published:** 2025-04-30

**Authors:** Dikeledi N. Malematja, Elizabeth M. Nkosi, Sanele E. Nene

**Affiliations:** 1 Department of Nursing, Faculty of Health Sciences, University of Johannesburg, Johannesburg, South Africa

**Keywords:** impact, insufficient, resources, quality of services, PHC clinic

## Abstract

**Background:**

Insufficient resources at healthcare clinics pose a serious problem, undermining the quality-of-service delivery and negatively affecting the patients as recipients of care and the staff as providers of care. The shortages often result in extended waiting periods, delayed implementation of nursing interventions, prolonged hospitalisation and the potential of increased nosocomial infections.

**Objectives:**

To explore and describe the impact of insufficient resources on the quality-of-service delivery at a primary healthcare clinic in Limpopo.

**Method:**

The study followed a qualitative, exploratory, descriptive and contextual design. Participants were selected using purposive sampling. Interviews were conducted until data saturation was reached. The interviews were audio-recorded, transcribed verbatim and analysed using Tesch’s eight-step method of data analysis. The study was steered by the Donabedian quality-of-care framework.

**Results:**

One central theme emerged, namely scarcity of healthcare resources, with three subthemes: (1) the impact of water shortage on the quality-of-service delivery, (2) the impact of staff shortage on the quality-of-service delivery and (3) the impact of medication shortage on the quality-of-service delivery.

**Conclusion:**

Insufficient resources negatively affect the quality-of-service delivery in healthcare. The provision of sufficient resources through collective managerial interventions is imperative to develop and implement measures to enhance the quality-of-service delivery.

**Contribution:**

This study may create awareness among the leadership about the challenges of the clinic. It may also facilitate the development and implementation of processes to provide the resources required to improve the quality-of-service delivery.

## Introduction

Many nations recognise universal healthcare as a right as it is linked to better health and lower disease rates (Guimarães, Lucas & Timms 2019). The *Constitution of South Africa* (1996) entrenches this right in Section 27, ensuring access to healthcare, including reproductive services and outlawing the denial of emergency care.

According to Abrahams, Thani and Kahn (2022), insufficient resources are a global healthcare challenge, especially in developing countries like South Africa. In South Africa, historical inequality has created unjust healthcare disparities, with Neely and Ponshunmugam (2019) identifying resource scarcity, transportation hurdles and nepotism as major barriers. Most South African communities struggle with unemployment and financial hardship and thus rely heavily on public healthcare services (Matlala et al. 2019). With more than 86% of the South African population receiving healthcare services from the government, literature estimates that about 8.5% of the gross domestic product is allocated to healthcare. This results in insufficient resources and public outcry about the shortage of medication, staff and other critical resources such as water and electricity (Mosadeghrad 2016).

Healthcare resources refer to all materials, including human resources, used to provide healthcare services (Ransom & Ollson 2017). When resources are scarce, it affects everyone. The staff becomes overwhelmed trying to make ends meet, patients feel neglected when staff step out to borrow or improvise to compensate for the unavailable resources, and the completion of nursing chores is delayed. Patients get frustrated, impatient and eventually lose trust in the healthcare system. This often boils over as increased patient complaints and negative reviews are sent to the media and to the Office of Health Standards Compliance (OHSC). Some patients’ families have reportedly threatened to start legal action (Doerine et al. 2022). Quality service delivery refers to the level of satisfaction of the clients in relation to the services rendered by the organisation (Abdullahi & Osman 2023). Previous studies have identified insufficient staff as a challenge, but despite numerous attempts to resolve it, no solution has been found. According to the World Health Organization (WHO 2019), statistics reveal fewer healthcare providers in Africa compared to Europe. For instance, Africa has 13 nurses and midwives per 10 000 population compared to 83 per 10 000 in Europe. This puts a strain on the existing human resources. For instance, nurses have to put in more effort to complete all their nursing interventions at their respective facilities. Mutshatshi and Munyai’s (2022) study highlighted that the scarcity of resources, particularly human resources, is a key factor in the ineffective delivery of 24-h services at health facilities nationally. Inspections conducted by the OHSC in 2015/16 identified several issues at South African healthcare facilities. This included, among other things, inadequate infrastructure, medication shortages and a significant staff shortage (Mazwai et al. 2018). Maphumulo and Bhengu (2019) affirmed that a shortage of staff remained one of the major weaknesses in the sub-Saharan Africa health system.

Barron and Padarath (2017:4) concurred that in South Africa, these weaknesses are aggravated by the unequal distribution of healthcare professionals between the private and public sectors, coupled with the unequal distribution of public sector health professionals among the provinces. This is consistent with the findings of Garcia-Vera et al. (2018).

South Africa is working towards universal health care (UHC), which would guarantee quality healthcare regardless of finances (WHO 2013). The current strategy involves rolling out a national health insurance programme to get closer to this ideal (Fusheini & Eyles 2016; WHO 2013). However, the clinic in question does not meet UHC standards or the WHO’s criteria for equitable, people-centred service delivery. The clinic operates for 12 h each day, from 07:00 to 19:00, and is not open at night because of staff shortages. Patients are occasionally sent away without receiving the necessary nursing interventions because of a shortage of much-needed resources. The closest 24-h hospital is approximately 64 km away. The local community bears the burden of transportation expenses to access healthcare services not readily available at this clinic.

The facility provides maternity services and is currently manned by three midwives working shifts. Because of staff shortages, patients requiring maternity and ante-natal care are sometimes transferred to other healthcare facilities for intervention. Patients who require midwifery intervention are occasionally redirected to hospitals because of a lack of water, which could jeopardise patient safety. This scarcity of water could lead to insufficient measures for infection control, thereby posing a potential threat to the safety of the patients and the staff at the clinic (World Health Organization / United Nations International Children’s Emergency Fund [WHO/UNICEF] 2019).

### Aim of the study

The aim of this study was to explore and describe the impact of insufficient resources on the quality-of-service delivery at a primary healthcare clinic in Limpopo.

### Setting

The study was conducted at a designated primary healthcare clinic in Limpopo province (Polit & Beck 2017). The clinic provides 12-h comprehensive care to approximately 3800 people in the nearby villages of Mamanyoha, Taolome and Hlohlokwe. The area, primarily rural, has a district hospital, Kgapane Hospital, which is situated about 64 km away. The staff compliment consists of one operational manager, one nurse clinician, two professional nurses and three enrolled nurses. The facility provides a wide range of services, including maternal and child health services, reproductive health services, testing and treatment for HIV and tuberculosis, management of non-communicable diseases, treatment for minor illnesses, maternity care, as well as screening, testing and vaccinations for coronavirus disease 2019 (COVID-19). Because of security concerns, the Limpopo Department of Health (DoH) reduced the clinic’s 24-h services to 12-h services in 2020, forcing the community to seek healthcare services from Kgapane Hospital outside the normal clinic operational hours.

## Research methods and design

The study followed a qualitative, exploratory, descriptive and contextual research design using 12 semi-structured interviews (Creswell & Creswell 2018; Kirkman, Hammarberg & De Lacey 2016). This design was suitable as it enabled the researcher to capture the responses of the clients and to gather unique information on the impact of insufficient resources on the quality-of-service delivery at the Mamonyoha primary healthcare clinic (PHC) in Limpopo (Gray & Grove 2020; Allison 2018). The study was steered by the Donabedian quality-of-care framework.

### Population and sampling

The target population of this study included all clients who use the services at the PHC clinic in Limpopo (Burns, Grove & Gray 2017). They were selected as they had information about the phenomenon being studied and were willing to share their experiences on the impact of insufficient resources. The sample was selected using a purposive sampling method (Creswell & Creswell 2018; Sharma 2020).

### Inclusion and exclusion criteria

The inclusion criteria were as follows: clients who had used the PHC clinic more than three times in the last year, who were between 18 years and 59 years of age, who were willing to share information with the researcher and who had voluntarily consented to participate in the study. Clients who had used the clinic fewer than three times in the preceding 12 months were excluded.

### Data collection

Data were collected at the PHC facility at Mamonyoha in Limpopo. The researcher conducted the recruitment of potential participants. An initial meeting was scheduled with the hospital manager as an information session to explain the purpose of the study. At this meeting, the researcher agreed that the smooth running of the clinic will not be affected by the recruitment and planned interview process. The study was advertised on posters posted at the clinic and the staff informed possible participants about the study during the clinic operational hours. Interested individuals were asked to contact the researcher or visit the facility, where more information on the study and objectives were shared. All participants who met the eligibility criteria and agreed to be part of the study were given information letters and consent forms to consent to interviews and audio-recording (Borovecki et al. 2018). The participants contacted the researcher individually to indicate their interest or to receive further clarification from the researcher. The sample of this study included clients who consented to partake voluntarily. The consent forms contained information on participants’ rights to withdraw from the study at any time without penalty. The researcher collected the consent forms before data collection started (Bhandari et al. 2020).

The participants chose the dates and times of the interviews depending on their availability. The venues were chosen to ensure privacy and had adequate lighting, comfortable chairs and a table (eds. Creswell & Poth 2018). The researcher used semi-structured interviews to obtain sound and trustworthy data that would successfully explore and describe the experiences of the participants. The participants were asked open-ended questions in Sepedi, the language most spoken by the clients of Mamanyoha. The interviews took place on Friday afternoons when the PHC clinic was not busy to prevent disruption or delays in scheduled facility visits. Those who were not available on Fridays chose other suitable days and times according to their availability.

The duration of the interviews ranged between 45 min to 60 min, and the central question that the researcher asked the participants was: ‘What is the impact of insufficient resources on the quality of care at the clinic’. Follow-up questions were based on the research purpose and participants’ responses. Observations were recorded as field notes during or immediately after the interviews. The researcher used various communication skills to gather as much data as possible. Probes included statements like: ‘Tell me more’; ‘is that all?’; ‘did you mean that?’ to summarise and capture the final responses without interruption. Nonverbal cues such as nodding the head (Holloway & Wheeler 2010) encouraged participants to elaborate and provide rich information in response to the research question. The data collection continued until data saturation, which was reached after 12 interviews. Two more interviews were conducted to confirm data saturation.

### Data analysis

The study relied on Tesch’s eight-step method of qualitative data analysis (Creswell 2014). The audio-recorded interviews were transcribed verbatim immediately after the interviews, translated into English and then analysed. This process involved reading all transcripts, selecting interesting documents, compiling a list of similar topics, clustering topics and coding the main points. Themes were formed by combining codes, and a final decision was made on the abbreviation for each theme. The researcher conducted the final analysis, summarised the key findings and outlined how they related to the study. An independent coder who is an expert in qualitative researcher and holds a master’s degree in nursing, followed the same steps of data analysis as the researcher to assist with the coding of the data. A consensus meeting was held between the researcher, the independent coder and the research supervisors, where agreement was reached on the central theme and subthemes emerging from the data.

### Ethical considerations

The study adhered to the ethics principles as indicated by Dhai and McQuoid-Mason (2020), throughout the study, namely autonomy, beneficence and non-maleficence and justice. The participants were afforded the right to participate voluntarily and gave their consent without coercion. The participants had the right to withdraw from the study at any time without penalty. The collected data, the audio-recordings and field notes were kept in a safe place and access was restricted to only the researcher, the supervisor and co-supervisor (Polit & Beck 2021).

Ethical clearance to conduct this study was obtained from the University of Johannesburg Faculty of Health Sciences Research Ethics Committee (No. REC-1339-2021). Permission to conduct the study was obtained from Limpopo Department of Health Research Committee and from the management at Mamanyoha primary health clinic. The participants received sufficient information on the study to ensure their understanding of the purpose and expectations and to enable them to consent voluntarily. The researcher looked out for any signs of emotional discomfort from the participants to avoid harm to their emotional well-being. The principle of justice was observed by ensuring that the participants were selected and treated fairly.

## Results

### Demographic profile of the participants

Data were collected from 12 participants, including five males and seven females. The ages ranged from 18 years to 59 years. Although most of the participants understood English and could respond using it, they all preferred to communicate and respond to the questions in Sepedi as their home language. The participants were asked open-ended questions in Sepedi, the language most spoken by the clients of Mamanyoha. An interpreter was present to interpret the interviews. Table 1 presents the demographic profile of the participants.

**TABLE 1:** Demographic profile of the participants.

Number	Gender	Ethnicity	Age (years)	Village
P1	Male	Bapedi	56	Taolome
P2	Female	Bapedi	28	Mamonyoha
P3	Female	Bapedi	43	Mamonyoha
P4	Female	Bapedi	51	Taolome
P5	Female	Bapedi	43	Hlohlokwe
P6	Female	Bapedi	32	Mamonyoha
P7	Female	Bapedi	28	Taolome
P8	Male	Bapedi	27	Hlohlokwe
P9	Male	Bapedi	56	Hlohlokwe
P10	Female	Bapedi	18	Mamonhoha
P11	Male	Bapedi	29	Taolome
P12	Male	Bapedi	33	Mamonyoha

*Source*: Malematja, D.N., 2023, ‘Perceptions of clients on quality of health service delivery at a primary healthcare facility in Limpopo’, Masters thesis, Department of Nursing Sciences, University of Johannesburg

One central theme and three subthemes emerged from the data. The main theme was the scarcity of resources. Subthemes included the impact of water shortages on the quality-of-service delivery, the impact of staff shortage on the quality-of-service delivery and the impact of medication shortages on the quality-of-service delivery. The themes are summarised in Table 2 and discussed. Field notes are presented following the quote from the study participants.

**TABLE 2:** Summary of themes and subthemes.

Main theme		Subthemes
1.	Scarcityof resources	1.1 The impact of water shortages on the quality-of-service delivery 1.2 The impact of staff shortage on the quality-of-service delivery 1.3 The impact of medication shortages on the quality-of-service delivery

*Source*: Malematja, D. N., 2023, ‘Perceptions of clients on quality of health service delivery at a primary healthcare facility in Limpopo’, Masters’ thesis, Department of Nursing Sciences, University of Johannesburg

### Scarcity of resources

The participants revealed that they resorted to traditional medicine because of the scarcity of resources at the clinic. Two of the participants alluded to poor hygiene and poor infection control measures at the clinic as there was no water for the staff to wash hands between patients. This was a concern as they were worried about the transfer of illnesses between clients. Some expressed their dissatisfaction and doubt on whether they should return for another visit for their chronic medication. This is confirmed by the following quotes:

‘I came here to collect my treatment; I cannot leave with other patients’ diseases because there is no water for the nurses to wash their hands.’ (Participant 11, 29-year-old male)

‘I waited for more than ten minutes for the nurse to bring water for me to drink my tablets. I am better off at home than here…I don’t know why I bothered … things are getting worse, but I do not blame the nurses. Where are the supervisors to witness this frustration?.’ (Participant 12, 33-year-old male)

A previous study by Nevhutalu (2016) confirmed dirty toilets and malfunctioning equipment such as blood pressure monitoring machines. Quality service delivery relies on adequate resources, and in the event of scarcity, the implications are disastrous as it becomes challenging for nurses to execute their duties efficiently (Jooste 2017). This is consistent with the findings of Nene et al. (2020).

### The impact of water shortage on the quality-of-service delivery

The participants expressed their frustrations with the water shortage and mentioned that it has a negative effect on the facility’s service delivery. They added their concern about being expected to present at a clinic for healthcare services without a critical resource such as water. In concurrence with the findings, the participants expressed themselves as follows:

‘After giving birth to my baby, I wanted to take a shower, but I was informed that the facility had no water. “I had to wait until I was discharged to bathe at home. It was very uncomfortable for me, imagine having to sleep with blood all over my body”.’ (Participant 7, 28-year-old female)

‘The toilets inside the facility were non-functional because there was no running water, toilets were not flushing, people had to use outside pit toilets and there was no water to wash their hands.’ (Participant 2, 28-year-old female)

‘The nurses touch different patients and only use sanitisers sometimes without washing their hands because there is no water at the clinic, sometimes I wonder how they treat patients with open bleeding wounds and those who are giving birth.’ (Participant 1, 56-year-old male)

‘We really had to compromise but remember if you do not have resources of treating the patient like water for instance, you will not give the total quality patient care.’ (Participant 7, 28-year-old female)

### The impact of staff shortage on quality-of-service delivery

Participants voiced their dissatisfaction about the shortage of qualified nursing staff at the facility. They mentioned that management should address and prioritise the provision of qualified PHC nurses as this will enhance the quality-of-service delivery. The following quotations from the participants verify the findings:

‘The community sometimes complains about pregnant women being turned away or referred to hospital because the nurse who is supposed to help them is sick or on leave. So those things lower the good service standards.’ (Participant 9, 56-year-old male)

‘Sometimes there are no midwives at night. Imagine what will happen to my daughter who is due to deliver any time from now? I am thinking of relocation to another village just till after the baby is born then we can return home. I waited for too long for this grandchild and cannot afford to lose him because we do not have nurses at our clinic at night.’ (Participant 3, 43-year-old female)

‘There is a shortage of staff because sometimes you find only one professional nurse on duty and when we ask, we are told that the shift has only two professional nurses and one is sick.’ (Participant 9, 56-year-old male)

### The impact of medication shortages on the quality-of-service delivery

Participants mentioned a shortage of medication at this facility and the subsequent negative effect on service delivery. The following quotes clarify this:

‘The last day when I brought my child for immunisation, I was told that the vaccines are out of stock, but they promised that they will order, and my child will get it on her next visit.’ (Participant 6, 32-year-old female)

‘Another issue is the shortage of medicine. We are sometimes denied treatment when we visit the facility because they say it is not available.’ (Participant 4, 51-year-old female)

‘During the day we are working, and we have our traditional medicines to treat ourselves. However, when we get back home, and the illness gets worse there are no tablets nor anything to help us. So, I will die at home instead of getting help … I think it is better to stay at work till I get better because, think about it … why go home to face neglect by a clinic that cannot help you? I cannot believe this.’ (Participant 8, 27-old-year male)

## Discussion of results

### The impact of water shortage on quality-of-service delivery

The findings of this study reveal that the participants experienced the absence of clean, safe water on numerous occasions when they arrived for their monthly review at the clinic. Others mentioned their dismay, disappointment and frustration at the lack of water on visiting the facility. The majority stated that they fear infections because of the lack of water. This was consistent with the findings of a study by Cronk and Bartram (2018) and National Department of Health (2011). The provision of quality healthcare should occur in a hygienically safe and clean area with an adequate supply of clean running water for the staff and patients. The absence of clean safe running water predisposes the clients and staff to viral, bacterial and fungal infections, which can complicate illness and lead to patients’ death, as testified by Ismail and Perovic (*The Conversation* 2023).

### The impact of staff shortages on the quality-of-service delivery

The participants confirmed that there is a serious shortage of staff at the clinic, which undermines the provision of care. This shortage leads to delayed caregiving. Nursing tasks like admissions and blood pressure monitoring are either incomplete or not executed at all. The waiting period is long as one nurse attends to numerous patients at any given time. The staff shortage affects the nurses negatively as they can spend less time with each patient, and they have an increased workload. The findings were validated by an earlier study by Rensburg (2021) and Lasater et al. (2021), which indicated that insufficient staffing could trigger a multitude of detrimental effects. Nene, Ally and Nkosi confirmed that the lower the numbers of required nursing staff, the higher the incidents of adverse events. The provision of adequate staff is necessary for the smooth running of a primary healthcare clinic. Without staff, there is no healthcare service delivery. The participants further explained that staff shortages have the potential to perpetuate a decline in the quality-of-service delivery, which is consistent with the findings of Kamndaya et al. (2014:581) and Nesengani et al. (2021).

The participants recommended the employment of more staff at the clinic to ensure that the waiting periods are less. This will ensure that those on duty do not burn out or become overwhelmed by the tasks to be performed. Staff shortages, longer waiting periods and delayed caregiving cause patients to leave the clinic and resort to home and traditional remedies. When the number of patients who use the clinic declines, the existing staff becomes demoralised and discouraged, resulting in increased resignations and a high rate of absenteeism, as affirmed by Lasater et al. (2021).

The healthcare system in South Africa is nurse-based, with the PHC facilities consulted most. Shortages of resources at these facilities can result in a decline in the quality-of-service delivery (Maphumulo & Bhengu 2019).

### The impact of medication shortages on the quality-of-service delivery

The participants emphasised the importance of providing sufficient medication at these healthcare facilities. The shortages can lead to increased patient complaints and escalated financial burdens for patients as they must travel to different facilities with alternative means of transport to receive their chronic medication. Some participants decided to use other clinics to get their medication despite the associated financial burdens. Participants recommended that the clinic management should consider additional healthcare funding to improve supply chain management to reduce the risk of medication shortages. It also underscores the importance of maintaining sufficient pharmacy staff, particularly by employing pharmacists or pharmacy assistants at all PHC facilities. This can help to alleviate the shortage of medication as sufficient qualified personnel can maintain control over the availability of medication and timeous reporting for replenishing. Modisakeng et al. (2020) confirmed the importance of a consistent supply of medication. According to the findings, patients die from curable diseases because of medication shortages. When a clinic is unable to meet the expectations of the patients, the consequences are disgruntled patients, leading to negative outcomes for the patients’ commitment to their treatment regime, including referrals and patient retention. This is consistent with the findings of Yenet, Nibret and Tegegne (2023).

The South African healthcare system is hampered by insufficient resources, from equipment to materials and human resources. A shortage of resources can damage the reputation of the healthcare system, as it may lead to a rise in patient complaints. This has been substantiated by Doerine et al. (2022). The level of health service provision at healthcare facilities is reliant on access to healthcare resources (Mosadeghrad 2016). The resources required for healthcare services encompass human resources, medication, medical devices and infrastructure (Ransom & Olsson 2017).

### Study limitations

The findings of the study are contextual in nature, specifically relevant to the service area of the Mamanyoha primary healthcare clinic, which serves mainly the villages of Taolome, Hlohlokwe and Mamanyoha. The results of the study may not be applicable to all clients of the Mamanyoha clinic. Some clients were hesitant to expose the inadequacy of the local chiefs and staff alike.

### Recommendations

Policymakers and healthcare administrators should invest in a reliable water supply system and initiate strategies to conserve water, including devising a backup plan for adequate, uninterrupted water supply. Underlying staffing issues such as resignations, retirements, chronic illness and mortality require collective collaboration with the DOH and relevant stakeholders. Potential solutions could involve investing in staff training and development to increase the healthcare workforce and improving working conditions and salaries to attract more individuals to the healthcare facility. Wellness services should be offered to promote employee well-being. Health education should be provided to the chiefs of the rural areas to educate them on preserving the available water. Hospital management teams should have systems to address water, sanitation and hygiene (WASH) malfunction or other related issues such as infrastructure maintenance. Further research is necessary to explore this phenomenon from the perspective of the healthcare providers. The negative effect of resource shortages in healthcare is a pressing issue that demands the immediate attention from policymakers and health management. Insufficient resources, which include human and material resources, have plagued the South African healthcare system for a decade and require prompt intervention to save the lives of patients and ensure quality service delivery (Nene et al. 2020).

## Conclusion

Shortages in healthcare are far-reaching and deeply damaging, affecting not only the quality of care to patients but also the financial stability and reputation of healthcare institutions. The scarcity of resources such as water, personnel and medication compromise the quality of care and undermine trust in the healthcare system. Staff shortages make the provision of quality services challenging as tasks are executed at a slower pace. Therefore, it is imperative for healthcare facilities to ensure the availability of necessary resources, which includes clean safe water, staffing, medication, medical devices and infrastructure, to deliver effective health services and maintain the reputation of the healthcare system. This will ultimately contribute to improved patient satisfaction and better health outcomes.
